# Effectiveness of temperament based therapy with support: a randomized controlled trial study protocol

**DOI:** 10.3389/fpsyg.2026.1734105

**Published:** 2026-04-02

**Authors:** Kristin Stedal, Ingrid Funderud

**Affiliations:** Regional Department for Eating Disorders, Division of Mental Health and Addiction, Oslo University Hospital, Ullevål, Oslo, Norway

**Keywords:** anorexia nervosa, eating disorders, family-based treatment, randomized controlled trial, TBT-S, temperament-based treatment, multi-family treatment

## Abstract

**Background:**

Temperament-Based Therapy with Support (TBT-S) is a neurobiologically informed, skills-based intervention for eating disorders in which individuals with anorexia nervosa (AN) and their supports participate together in a multi-family group format over five consecutive days. The program is delivered as an adjunct to treatment as usual (TAU). Although prior studies have demonstrated the feasibility, acceptability, and promising outcomes of TBT-S, its effectiveness remains uncertain, as no previous studies have included a control group. This study aims to evaluate the effectiveness of TBT-S by determining whether adding TBT-S to TAU is superior to TAU alone.

**Methods:**

In this multicenter randomized controlled trial (RCT), 120 adults with a diagnosis of AN or atypical AN (AAN) will be randomized to receive either TBT-S in addition to TAU or TAU alone. Individuals with AN/AAN and their supports will complete assessments before and after the TBT-S treatment week, and at 3- and 12-month follow-ups. The primary outcome is change in eating disorder symptomatology from baseline to the 3-month follow-up. Secondary outcomes include changes in quality of life, psychological health, body mass index (BMI), and health care utilization, as well as changes in quality of life and caregiving experience among supports.

**Discussion:**

If TBT-S is found to be effective in reducing eating disorder psychopathology, it may represent a valuable and scalable adjunct to standard treatment for adults with AN/AAN.

## Introduction

Anorexia nervosa (AN) is a psychiatric disorder associated with substantial functional impairment. It is characterized by restriction of energy intake leading to significantly low body weight, an intense fear of gaining weight or persistent behaviors that interfere with weight gain, and a disturbance in the experience of body weight or shape; associated features may include preoccupation with food, repetitive body checking, compulsive exercise, and marked anxiety related to eating (American Psychiatric Association, 2022). The mortality rate for individuals with AN is significantly greater than for other psychiatric in-patients and for the general population (Miskovic-Wheatley et al., 2023; Walker et al., 2015).

Because of its early age of onset, chronicity and level of disability, AN is responsible for an inordinate burden upon the health services (Wentz, 2023). Chronic courses of AN are common and typically involve severe long-term consequences with several admissions and readmissions to hospital (Muratore and Attia, 2021). For adolescents, family-based treatment has been shown to be associated with high rates of remission and recovery (Monteleone et al., 2022) and is recommended as the first-line treatment approach (NICE, National Institute for Health and Care Excellence, 2020). For adults, there is growing evidence pointing to the usefulness of behaviorally focused psychotherapies, and commonly utilized approaches include Cognitive Behavior Therapy-Enhanced (CBT-E), Specialist Supportive Clinical Management (SSCM), and Maudsley Anorexia Nervosa Treatment for Adults (MANTRA) (Russell et al., 2023). However, there are currently no specific treatment approaches which show clear superiority in the treatment of adults with AN (Costandache et al., 2023). Adults with AN have often had a longer duration of illness, have higher relapse rates and are less responsive to treatment (Costandache et al., 2023; Solmi et al., 2024). Hence, it has been stated that “additional studies in this population are crucial, especially if they might target mechanisms underlying AN or identify core treatment components that help individuals make optimal improvements” (Muratore and Attia, 2021, p. 91).

Temperament based therapy with support (TBT-S) is a neurobiologically-informed skills-based intervention for AN where individuals with AN and their supports (e.g., parent(s), partner, other family member and/or friend) participate together in a multi-family format (Hill et al., 2022). TBT-S directly targets eating disorders symptoms by providing a framework for understanding these behaviors and how to manage them. This framework builds on research from cognitive neuroscience showing that individuals with AN often exhibit heightened cognitive control and rigidity, reduced sensitivity to reward (particularly food-related reward), and altered insula and striatal functioning, which is believed to contribute to persistent eating disorder behaviors, despite negative physiological and psychological consequences (Wierenga et al., 2018; Kaye et al., 2015). It integrates neurobiological psychoeducation and structured skills training to enhance symptom management through a strength-based framework. Rather than pathologizing temperament traits commonly associated with AN, TBT-S leverages these characteristics as adaptive assets in the recovery process and allows for an individually tailored treatment approach—based on a manualized framework (Hill et al., 2022). The TBT-S tools are designed to be action-oriented and trait-aligned, so clients can select tools that build on their specific strengths and help reroute from ED symptom cycles. For example, the “Stop, Reboot, Reroute” tool is a step-by-step method for interrupting eating disorder thoughts/behaviors and choosing a more adaptive action. The TBT-S toolbox draws from other eating disorder treatment approaches and is intended as a selection of strategies which could be helpful for managing symptoms while aligning with the individual’s temperament traits. Because supports learn the same tools, they can use these to help coach during meals, distress, and high-risk moments. Building on research showing that family therapy—and possibly in particular multifamily therapy—is helpful in treating individuals with AN (Wierenga et al., 2018), supports are included and have an active role throughout the treatment week. This can aid in reducing isolation and increases accountability—as well as helping to translate TBT-S tools into daily life and thereby sustain treatment gains over time.

TBT-S is an intensive treatment delivered over five consecutive days for a total of approximately 35-40 hours - as an adjunct to treatment as usual (TAU) (Wierenga et al., 2018). It has demonstrated good feasibility, acceptability and outcomes in uncontrolled trials (Wierenga et al., 2018; Knatz Peck et al., 2021; Stedal et al., 2023; Skoog et al., 2025). After TBT-S, 31% of adults with AN were fully or partially remitted, and at 6 months follow-up, 61% of the participants were considered partially or fully remitted (Wierenga et al., 2018). Similarly, for young-adults, full or partial remissions were reported for 39% post TBT-S treatment, and for 56% at 1 year follow-up (Knatz Peck et al., 2021). Studies have also demonstrated improvements in eating disorder psychopathology and clinical impairment, both immediately after receiving TBT-S (Stedal et al., 2023) and at six (Wierenga et al., 2018) and 12 month (Knatz Peck et al., 2021; Skoog et al., 2025) follow-ups. However, these previous studies are limited by a lack of control group. Thus, there is a need for fully powered randomized controlled trials (RCTs) with longer follow-up periods to further establish the effectiveness of TBT-S.

### Aim and objectives

The primary objective of the study is to examine the effectiveness of TBT-S when applied in conjunction with TAU. Specifically, we will measure whether TBT-S in addition to TAU will be more effective than TAU alone in reducing eating disorder psychopathology. We hypothesize that participants receiving TBT-S in addition to TAU will show significantly greater reduction in eating disorder psychopathology from TBT-S pre-assessment to 3-month follow-up, compared to controls.

Our secondary objectives will explore whether participants receiving TBT-S in addition to TAU show reduced clinical impairment, increased quality of life and lower utilization of health care services compared to participants receiving TAU only, at 3-and 12-month follow-ups. Secondary objectives also include the comparison of support experiences in terms of quality of life, psychological wellbeing, carer accommodation and expressed emotional experience of caring for a person with an ED.

## Method

### Study design

**Figure 1:**
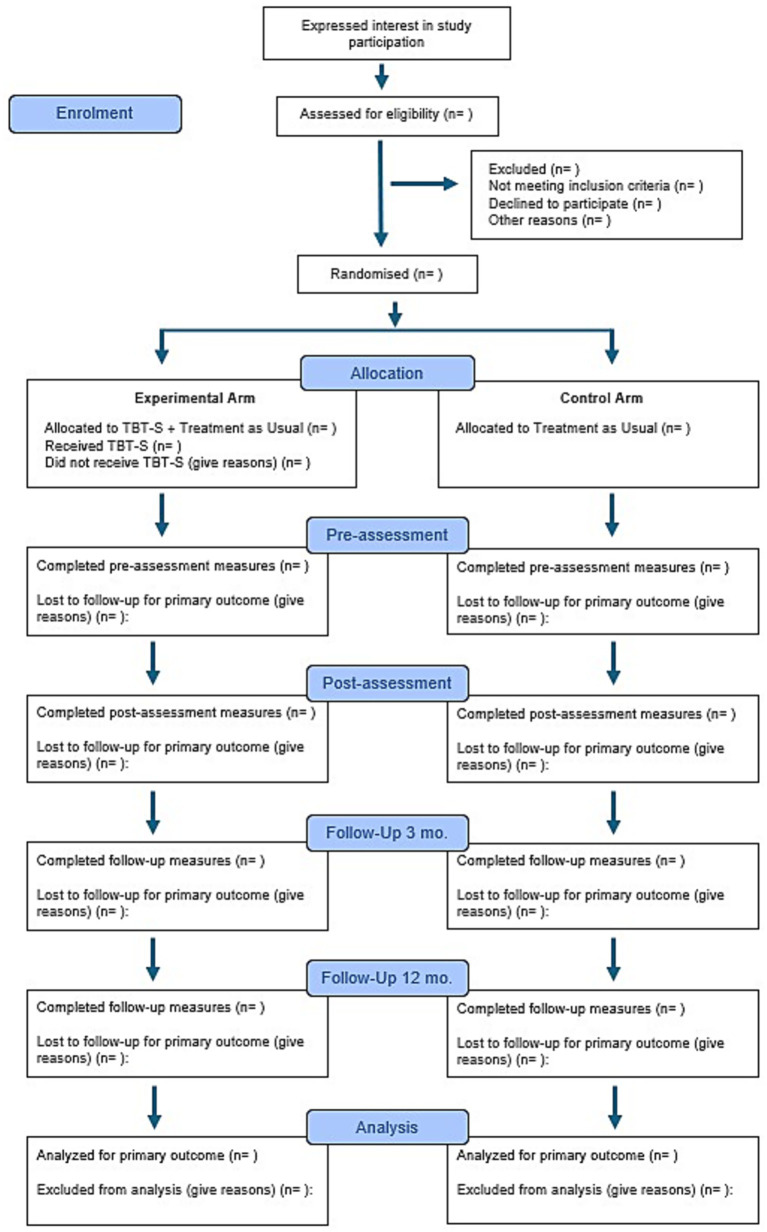
Consolidated Standards of Reporting Trials (CONSORT) flow chart.

We will conduct a non-blinded, randomized, controlled, parallel-group, superiority trial comparing (i) TBT-S plus TAU to (ii) TAU only. Similar control conditions have been used in previous trials of short-term interventions for eating disorders (e.g., Godart et al., 2012; Gowers et al., 2010; Quiles et al., 2025). Four main assessments will be conducted: pre-assessment, post-assessment, and 3- and 12- months follow-up. Eating disorder service utilization during the preceding 3 months will be assessed at pre-assessment and at 3-, 6-, 9-, and 12-months follow-ups. See Figure 1 for Consolidated Standards of Reporting Trials (CONSORT) flow chart (Hopewell et al., 2025) of the trial. The project will be arranged as a multisite study with two collaborating units (Regional department for eating disorders (RASP) at Oslo university hospital and the Department of eating disorders (RAS) at Haukeland university hospital). The collaborating clinics offer TBT-S as part of their standard clinical practice. In accordance with the treatment manual (Hill et al., 2022) there are no requirements regarding the specific time point during the ongoing treatment at which TBT-S should be introduced; clinicians can integrate TBT-S flexibly into ongoing therapy. Thus, the timing of TBT-S within TAU is individualized rather than standardized and depends primarily on practical considerations, including participants’ ability to take time off for the 5-day intervention and the scheduling of TBT-S weeks at the treatment sites (4–6 times per year). Multiple participants are assigned to each TBT-S week before being randomized to receive TBT-S plus TAU or TAU only. Participants randomized to TAU only complete assessments at the same time-points as those randomized to the corresponding TBT-S week, ensuring comparability across study arms.

The first author (Dr. Stedal) is a licensed TBT-S trainer who initiated the intervention in Norway in 2018, conducted the feasibility study in 2023 (Stedal et al., 2023), and is the principal investigator (PI) of the current effectiveness trial. The selected assessment measurements, timeframes, data platforms, recruitment procedures, sample size calculation, and statistical analyses build on the experiences gathered from the feasibility trial. The study has been approved by the Norwegian Regional Committees for Medical and Health Research (REK, 728279), the Data Protection Officer at Oslo University Hospital (PVO, 24/11463) and registered at ClinicalTrials.gov (NCT06497101). Any modifications to the protocol will be reported on clinicaltrials.org, and if relevant, submitted to the Regional committees for Medical and Health Research.

### Participants, eligibility criteria and recruitment

Adults receiving treatment for AN or atypical AN (hereafter AN/AAN as a shorthand to signify that both conditins are being discussed as a singular diagnostic entity) at a local psychiatric center and/or from other qualified treatment provider could be eligible to participate in the study. We will utilize similar recruitment procedures as employed in our TBT-S feasibility study (Stedal et al., 2023). An information pamphlet and website have been designed to facilitate recruitment, complemented by targeted advertisements on social medial platforms. Individuals express interest by sending an e-mail to the TBT-S mailbox (tbts@ous-hf.no) hosted by Oslo University Hospital or by filling out an online form which leads to a screening call to determine eligibility.

*Inclusion criteria:* Age ≥18, body mass index (BMI) ≥ 15, currently receiving outpatient treatment for AN/AAN; self-reported medically stable (confined to physical criteria), have a minimum of one support person who can participate in the study.

*Exclusion criteria:* Intellectual developmental disorder or intellectual disability; psychotic disorder or other psychopathology that may affect or prevent participation in the study; diagnosed alcohol or other substance use disorder within 3 months prior to study initiation; ongoing inpatient treatment at the time of study enrollment (participants currently in inpatient care must be discharged and engaged in outpatient treatment before participating in the TBT-S week).

If criteria are fulfilled, informed consent from all study participants, including supports, will be obtained on an encrypted study platform (www.nettskjema.no) requiring identification and two-step authentication.

### Strategies to promote participant retention

During the informed consent process, participants with AN/AAN will be informed that they will receive a gift card upon study completion, and of the possibility to receive TBT-S after the 3-month follow-up if randomized to the control condition. Supports will be informed that they are entered into a lottery to win one of 10 gift cards. To enhance participant retention, small tokens baring the study logo will be sent to participants at two time points: (1) A tote bag upon entering the study and (2) a notebook approximately halfway through the study. At each assessment point, Viedoc will automatically notify participants via e-mail or text message that the questionnaires are available for completion. If participants do not complete the questionnaires, two reminder notifications will be sent prior to the closing of the questionnaire period.

### Randomization and concealment

Included participants will be randomized using Viedoc, an online data collection platform hosted by Oslo University Hospital, Norway, approved by the data protection department at Oslo University Hospital, and monitored by a third party, the Clinical Trials Unit at Oslo University Hospital. This solution is adapted for randomization and gathering participant-reported data via PC, mobile or tablet. The Clinical Trials Unit has generated the randomization sequence in Viedoc which is stratified by treatment location and balanced by being built up of blocks of 2, 4 and 6 occurring in random order. Participant data will be identified by their assigned ID number rather than by name, the Norwegian identifier list will be stored in MedInsight, a Regional committees for Medical and Health Research approved digital participant list solution. All personal data will be stored on encrypted servers, requiring VPN-connection and/or two-step authentication (digital data) in accordance with data management guidelines from regional and local authorities.

Dates for TBT-S treatment weeks will be announced during advertisements so that participants can declare interest and availability for specific treatment weeks in advance of randomization. Allocation to TBT-S treatment groups (specific weeks) is based on the order in which participants declare interest and availability. Due to the nature of the intervention of this study, blinding of participants is impossible. Assessors are also not blinded, but are not included in the delivery of the intervention.

### Interventions

#### Temperament based therapy with support (TBT-S)

Participants in the intervention arm will receive 5 days of TBT-S in addition to ongoing TAU. TBT-S is administered to three to six adults with AN/AAN together with up to four supports each. As described in the TBT-S treatment manual (Hill et al., 2022), the treatment structure consists of five consecutive days of treatment, up to seven h each day. TBT-S is delivered in a multifamily format where both individuals with AN/AAN and supports are included in all the following five treatment strategies: (1) *Neurobiological psychoeducation*. Psychoeducation helps participants recognize how AN could be understood as a biologically influenced illness, emphasizing temperament traits (e.g., anxiety, harm avoidance, altered reward processes) and mechanisms that may be involved in maintaining eating disorder symptoms; (2) *Experiential learning activities addressing eating disorder neurobiology and traits*. The experiential activities are developed to aid participants experience how traits such as cognitive inflexibility can be experienced, how to maneuver toward recovery and how supports can be of help and, importantly, they help provide insight through action rather than discussion alone; (3) *Client and support skills training*. The skills training aspect of TBT-S teaches practical, trait-aligned tools to respond more effectively to eating disorder behaviors and to ensure consistent use of skills for recovery in daily life; (4) *Meal coaching*. Participants practice skills in real time together with supports and clinicians to help tolerate distress, reduce rituals and reinforce adaptive behaviors pre, post and during meals; (5) *TBT-S behavioral agreement*. The behavioral agreement is an individually tailored written plan, developed collaboratively by the participant with AN/AAN, their supports and the clinical team. It clearly outlines recovery-related behaviors and the role of supports. The agreement should help with accountability and provides a structure to facilitate consistent implementation of treatment strategies (Hill et al., 2022). Drop-out rates will be tracked systematically. We have previously translated and adapted the TBT-S material to Norwegian and assessed its feasibility and acceptability with Norwegian participants (Stedal et al., 2023).

#### Treatment as usual (TAU)

All participants will continue the treatment they receive when entering the study. TAU is defined as any treatment the participants receive for their AN/AAN other than TBT-S. Thus, TAU will vary and could include a combination of both psychotherapy and pharmacologic treatments. Participants in the control condition will differ from participants in the treatment condition in that they will not receive TBT-S in addition to TAU. In line with the recommendations by Petersson et al. (2023), we will give a detailed description of TAU, including a description of the content, and referring to clinical guidelines. At pre-assessment, and at 3-, 6-, 9- and 12 months follow-up assessments, participants will answer a questionnaire about the treatment they have received for their eating disorder in the last 3 months. The questionnaire includes items about level of treatment (e.g., outpatient psychiatric, inpatient psychiatric, general practitioner, and private vs. public), type of treatment (e.g., individual therapy, group therapy), intensity of treatment (e.g., number for days, sessions), and about whether and what pharmacological treatment the patient has used.

### Therapist competence and adherence

The two collaborating eating disorder clinics have a team of trained TBT-S clinicians. The TBT-S therapist trainings are structured in a three-tier manner, outlined on https://tbtstraining.com/training-levels/. The PI will provide training of untrained staff, as well as supervision of both clinics throughout the project period. At each site, TBT-S treatment weeks will be rated for intervention fidelity and therapeutic competence using checklists developed specifically for the study. Supervision based on these ratings will be provided to the clinicians if needed. Staff requirements for TBT-S are three clinicians, one dietician and one medical staff (Hill et al., 2022).

### Safety monitoring and adverse events

In cases where suicidal ideation is reported during the pre-assessment interviews, the interviewer will advise the patient to promptly consult their current eating disorder therapist or treatment provider. When participants register adverse events—defined as admittance to a somatic care unit or a psychiatric intensive care unit—in Viedoc, they will be prompted to provide additional information. At the same time, an automatic notification will be sent to the PI. These responses will be assessed by the project group every 6 months, and reviewed as to whether or not the incident could be caused by participating in the study. This assessment will be scored as: Unlikely, Somewhat Likely, Likely, Very Likely.

### Measurements

Table 1 provides an overview of interviews and self-report assessments at the different time points. Within 14 days before the start of a TBT-S-week, clinician-administered interviews will be conducted, and pre-assessment forms will be completed in both treatment arms. Post-assessments will be completed within 12 days after the last day of the TBT-S week and follow-up assessments within a 20 day period centered at 3-, and 12 month follow-ups. Use of health care services will be assessed at pre-assessment and at 3-, 6-, 9-, and 12-months follow-ups. The primary endpoint will be the group difference in change in eating disorder psychopathology (Eating Disorder Examination Questionnaire [EDE-Q (Fairburn and Beglin, 1994)] Global score) from pre-assessment to 3- month follow-up. The Norwegian version of EDE-Q has shown satisfactory psychometric properties (Rø et al., 2015; Reas et al., 2011). Interviews performed at pre-assessment are the Mini International Neuropsychiatric Interview [M.I.N.I. 7.0.2 (Sheehan et al., 1998)] and the Eating Disorder Assessment for DSM-5 [EDA-5 (Sysko et al., 2015; Dahlgren et al., 2020)] which are administered to assess comorbidities and confirm the AN/AAN diagnosis self-reported by the patient. When travel time to the main recruitment site (Oslo university hospital) allows for in-person assessments, participants are invited to fill out the forms on site, where weight and height will also be measured and registered. All other participants will complete the self-report assessments remotely and self-report weight and height. The M.I.N.I. and EDA-5 interviews of these patients will be conducted via a secure digital video platform. The main tools used for assessment are all previously validated and well-established measures (Akoury et al., 2018; Bouwmans et al., 2013; Cejalvo et al., 2025; Dambi et al., 2018; Feng et al., 2021; Fowler et al., 2014; Gratz and Roemer, 2004; EuroQol Group, 1990; Hibbs et al., 2014; Hoefman et al., 2013; Håseth et al., 1990; Osman et al., 2012; Pedersen et al., 2022; Sepulveda et al., 2009; Spielberger, 1970; Timman et al., 2015; Wiedemann et al., 2002; Winkler et al., 2019). Utilization of health care services and data on resource use will be collected using the self-reported Treatment Inventory of Costs in Patients with psychiatric disorders (TIC-P) questionnaire (Bouwmans et al., 2013; Timman et al., 2015). To ensure a detailed characterization of TAU, additional items on eating disorder service utilization have been added. These include whether the participant is currently receiving ED treatment, level of care and treatment intensity, as well as type of therapy and treatment modality. This information on service utilization together with the EuroQoL 5D [EQ-5D (Feng et al., 2021; Group, 1990)] and the Care-related Quality of Life instrument [CarerQol-VAS (Cejalvo et al., 2025)] will be used to assess the potential cost-effectiveness of TBT-S. Cost will be examined from both the healthcare provider as well as the societal perspective. Norwegian translations of all measures will be used. Two of the measures (Accommodation and Enabling Scale for Eating Disorders and Eating Disorder Quality of Life Scale) have not been validated in Norwegian. TBT-S attendance will be assessed at post-assessment (TBT-S plus TAU group only) using a study specific question added to the Client Satisfaction Questionnaire, asking participants to report how many of the five TBT-S treatment days they attended. At the 3- and 12- month follow-ups, adherence to TBT-S treatment recommendations will also be assessed using self-report measures, including the extent to which participants who received TBT-S have utilized the treatment tools provided during the treatment week.

**Table 1:** Overview of study timeline and measures.

		Timeline						
Assessments		Pre-assessment	TBT-S week*	Post-assessment	3 months	6 months	9 months	12 months
BMI^†^		x			x			x
Clinical interviews								
MINI		x						
EDA-5		x						
Questionnaires (pt)								
EDE-Q		x		x	x			x
CIA		x		x	x			x
EQ-5D		x			x			x
Carer QoL-VAS		x		x	x			x
Motivation VAS		x		x	x			x
EDQLS		x			x			x
MSPSS		x			x			x
DASS		x			x			x
STAI		x		x	x			x
CSQ-8				x				
DERS-SF		x		x	x			x
TIC-P		x			x			x
Health Services for ED		x			x	x	x	x
Treatment Adherence					x			x
Questionnaires (su)								
DASS		x			x			x
AESED		x			x			x
FQ		x			x			x
EQ-5D		x			x			x
Carer QoL-VAS		x			x			x

Note: TAU, Treatment as usual; TBT-S, Temperament based therapy with support; BMI, Body Mass Index; MINI, Mini International Neuropsychiatric Interview; pt, patients; su, supports; EDA-5, Eating Disorder Assessment for DSM-5; EDE-Q, Eating Disorder Examination Questionnaire; CIA, Clinical Impairment Assessment; EQ-5D, EQ-5D health-related quality of life questionnaire; QoL, Quality of life; VAS, Visual analogue scale; EDQLS, Eating Disorder Quality of Life Scale; MSPSS, Multidimensional Scale of Perceived Social Support; DASS, Depression Anxiety Stress Scale; STAI, State–Trait Anxiety Inventory; CSQ-8, Client Satisfaction Questionnaire; DERS-SF, Difficulties in Emotion Regulation Scale Short Form; TIC-P, Treatment Inventory of Costs in Patients with psychiatric disorders; ED, eating disorders; AESED, Accommodation and Enabling Scale for Eating Disorders; FQ, Family questionnaire. *Only participants in intervention group. †Height and weight of participants at the main recruitment site will be measured when travel time allows for in person assessments.

#### Implementation fidelity and therapists’ competence

The five key treatment components of TBT-S will be rated for degree of fidelity (10-point scale from 0 = No adherence; the section was skipped to 10 = Perfect; the treatment component was presented as advised in the manual). Therapist competence will be rated with 12 items (e.g., therapist delivering neurobiological lectures express ideas clearly and at an appropriate pace) using a 100-point scale with anchors for each item (e.g., 20 = Poor; therapists are difficult to follow and lecture proceeds at an uncomfortable pace, 100 = Superior; therapists are unusually articulate and express ideas in ways that all group members understand; perfect pace).

### Sample size calculation

The sample size calculation is conducted based on the primary outcome, global EDE-Q score at 3-month follow-up. It is informed by data from a feasibility study conducted by the authors (Stedal et al., 2023), in which the *SD* of the change in global EDE-Q from baseline to post-treatment was 0.87. A between-group difference in global EDE-Q change of 0.44 was assumed, based on the study by Tregarthen et al. (2019), who defines 0.5*SD as a clinically relevant difference in change from baseline, corresponding to a medium effect size (Cohen’s *d* = 0.5). In a two-sided test of superiority with *α*-level = 0.05 and 80% power, a total sample size of 126 is required. Participants are allocated to study arms in a 1:1 ratio. To account for the observed correlation (*p* = 0.732) between baseline and 3-month follow-up as reported in our previous feasibility study (Skoog et al., 2025), an ANCOVA framework is employed in the sample size calculations using raw EDE-Q global score at 3-month follow-up while adjusting for baseline. Thus, the number of participants is multiplied by the factor (1 + *p*)/2. Applying the correlation *p* = 0.732, the multiplicative factor is (1 + 0.732)/2 = 0.866. This results in an estimated required sample size of 110 participants. Assuming 10% attrition rate, we will include a total of 120 participants.

### Statistical analysis

Primary analyses will be conducted on the *Full Analysis Set* (FAS), defined as all participants who were randomly assigned to a treatment arm and who provided, at minimum, the following pre-assessment data; for individuals with AN/AAN, the EDE-Q; and for supports, at least one completed questionnaire, irrespective of the measure. As a sensitivity analysis for the primary outcome, the primary endpoint analysis will also be conducted on the *Per-protocol* (PP) population. In the TBT-S + TAU arm, the PP population will comprise those in the FAS who attended at least four of the five TBT-S days. In the TAU arm, the PP population will be identical to the FAS, as no additional intervention is delivered. Per protocol analyses will be adjusted for the time interval between randomization and pre-assessment.

The superiority of TBT-S plus TAU over TAU alone for the primary outcome will be analyzed using a linear mixed model with the 3-month EDE-Q global score as a continuous outcome, with baseline EDE-Q, treatment arm and study site, Time (factor) and Time (factor)*treatment arm interaction as fixed effects and subject ID as random effect. Secondary outcomes will be analyzed using linear mixed models in which baseline value, treatment arm, study site, time and time*treatment arm-interaction are included as fixed effects, and subject ID is included as random effect. The use of mixed linear models will allow for including in the analyses also participants that do not complete all assessments. As participants in the TAU alone condition may opt to receive the intervention after their 3‑month assessment, a proportion of TAU alone participants will undergo post‑3‑month treatment crossover. Analyses that include 6‑, 9‑, or 12‑month follow‑up will therefore incorporate analytic strategies appropriate for partial crossover. Further details of the statistical analyses will be specified in a statistical analysis plan, which will be completed before the database is locked and analyses commence.

### Patient and public involvement

A participant representative from a Norwegian eating disorder user organization (Rådgivning om Spiseforstyrrelser, ROS) is a member of the project group and will receive authorship credit on any publications where the Vancouver recommendations (International Committee of Medical Journal Editors, 2025) for co-authorship are met. ROS shows their support to the study by providing information about the project to their members and the public through their channels. Results from the trial will be disseminated to the public through open access scientific publishing and via presentations—both at scientific conferences as well as for the general population through media and user organizations.

## Discussion

This study will be the first controlled trial evaluating TBT-S and represents an important step in establishing treatment effectiveness. If TBT-S in conjunction with TAU demonstrates superior outcomes compared to TAU alone, it may warrant consideration as a standard adjunctive treatment for adults with AN. TBT-S provides adults with AN/AAN with an opportunity to actively involve family members or other support persons in their treatment. While most clinical guidelines recommend family-based treatment for children and adolescents with eating disorders (Benedictson et al., 2025), few structured options are available for adults who wish to include their support network (Baudinet and Eisler, 2024). Importantly, TBT-S may also help address the needs of family members who seek to understand the illness and learn how to provide effective support. This aligns with the advocacy of eating disorder user organizations, which strongly emphasize the inclusion of family members in treatment.

Participants included in the study will receive either TAU alone, or TAU in conjunction with TBT-S. Although this is the first time TBT-S is assessed in an RCT, previous studies have demonstrated that it is a highly endorsed treatment by both individuals with an eating disorder and supports, and have indicated that it could help reduce eating disorder symptoms (Wierenga et al., 2018; Knatz Peck et al., 2021; Stedal et al., 2023; Skoog et al., 2025). Notably, qualitative reports from participants indicate that some of the most helpful elements of TBT-S were; (i) how it improved the relationship between the individual with an eating disorder and their support, (ii) how their knowledge and understanding of eating disorders increased, and, (iii) how they gained more hope for the future (Stedal et al., 2024). These are all elements which are difficult to quantify and measure with standardized questionnaires, but nonetheless could be important for improving quality of life. Or, as stated by Hay and colleagues “Treatment trials need to move beyond targeting core eating disorder pathology (primarily weight restoration) and examine effectiveness in minimizing harm and reducing personal and social costs of chronic illness, such as medical morbidity, burden on health services, quality of life, depression and isolation (Hay et al., 2012, p. 1143).” Therefore, in this RCT we have ensured to also include measures of quality of life, anxiety and depression—also for the supports, and our 12-month health economy analysis will provide information on burden on health services. Although these are not primary outcome measures, they will undeniably be imperative to assess the impact TBT-S might have on these important variables.

The study has some potential limitations which are worth noting, some of which could impact the ability to interpret potential improvements. For example, the participants in the TBT-S arm will receive 35–40 h of additional treatment which is not controlled for in the control group. Thus, it will be uncertain if any improvements in the TBT-S group could be attributable to the increased dose of treatment, and/or non-specific elements in the TBT-S group (e.g., placebo, peer-support, behavioral activation). Nevertheless, this RCT marks an important first step in deciphering whether TBT-S in addition to TAU could improve treatment outcomes, and future studies might wish to include a comparison group program of similar intensity, with as little overlap as possible with TBT-S, thereby increasing the chances of better attributing any changes to TBT-S. Further, being allocated to TAU could be discouraging for some participants, increasing the risk of drop-out for this group. In addition, currently there are no other clinics in Norway offering adult TBT-S for AN/AAN. However, this could change over time and there is a risk that the TAU group gets tainted by TBT-S from a treatment provider not included in the study. Travel costs are covered through, a public service that reimburses travel expenses for healthcare treatments and clinical trials, and most employers will provide either paid or unpaid leave to receive healthcare. However, some eligible participants may still be prevented to participate in the study because they are not able to take time off work to attend TBT-S. Furthermore, the power analysis were based on our feasibility study with a comparable population. Even so, since both groups in this RCT will be receiving active treatments, there is a risk that potential differences between groups could be obscured by varying treatment settings and intensities, which would be expected to achieve reasonable effect sizes on their own. In addition, the timing of TBT-S within participants’ ongoing treatment is not standardized. As TBT-S is designed to be flexibly integrated into routine clinical care, participants may receive the intervention at different points in their treatment trajectory, which could contribute to heterogeneity in treatment response and introduce potential floor or ceiling effects. Finally, the lack of baseline assessment before randomization could result in the study’s pre-assessment being biased by the randomization. We will perform analyses of pre-assessments to check that the randomization produced equivalent groups. Any differences will be controlled for.

## Conclusion

As the first controlled trial of TBT-S, the outlined study will be a called for step in elucidating the effectiveness of this intensive 5-day treatment. This study is also the first to provide health economic and quality of life analyses of TBT-S outcome, an often neglected factor in treatment assessments. Findings from this trial have the potential to inform clinical practice and policy by clarifying the value of integrating TBT-S as an adjunctive intervention within standard care for adults with AN/AAN.

## Current trial status

Inclusion started in December 2024 and is expected to end December 2027. At the time of manuscript submission, 31 participants had been randomized. Analysis of data and reporting results will commence when primary endpoint data collection is completed.

## Data Availability

The original contributions presented in the study are included in the article/supplementary material, further inquiries can be directed to the corresponding author.
